# Supplementary material for: Assessment of leaf-litter invertebrate biodiversity using high throughput sequencing
Source: PLoS One. 2026 Aug 20;21(8):e0347811. doi: 10.1371/journal.pone.0347811 (PMC13492814; doi:10.1371/journal.pone.0347811)
Supplement: Supplementary file 1 [file pone.0347811.s001.docx]

S1 Table. Total DNA extraction yield for each of the three extraction protocols. (ps) DNeasy PowerSoil Pro; (bt) DNeasy Blood and Tissue; (zm) Quick-DNATM Plant/Seed Mini.

| **Protocol** | **Sample** | **Field** | | | | | | **Forest** | | | | | |  | **Negative control** |
| --- | --- | --- | --- | --- | --- | --- | --- | --- | --- | --- | --- | --- | --- | --- | --- |
|  |  | **1** | **2** | **3** | **4** | **5** | **1 - 5** | **1** | **2** | **3** | **4** | **5** | **1 - 5** | **Mean** |  |
| **DNeasy PowerSoil Pro** | **Weight (g)** | 0.216 | 0.32 | 0.272 | 0.289 | 0.29 | 0.263 | 0.274 | 0.266 | 0.284 | 0.279 | 0.27 | 0.278 | 0.275 | 0 |
|  | **Concentration (ng/µL)** | too high^*^ | 24.1 | too high^*^ | 36.7 | 27.2 | too high^*^ | 0.332 | 1.19 | 1.72 | 7.9 | 1.69 | 0.073 | 11.212 | too low^*^ |
| **DNeasy Blood and Tissue** | **Weight (g)** | 0.031 | 0.022 | 0.026 | 0.026 | 0.022 | 0.03 | 0.025 | 0.022 | 0.026 | 0.022 | 0.022 | 0.024 | 0.025 | 0 |
|  | **Concentration (ng/µL)** | 4.69 | 2.99 | 12.4 | 5.48 | 3.7 | 7.34 | 0.25 | 0.57 | 0.96 | 1.75 | 0.36 | 0.59 | 3.423 | too low^*^ |
| **Quick-DNA Plant/Seed Mini** | **Weight (g)** | 0.147 | 0.146 | 0.124 | 0.147 | 0.13 | 0.14 | 0.144 | 0.147 | 0.141 | 0.142 | 0.12 | 0.147 | 0.139 | 0 |
|  | **Concentration (ng/µL)** | 35 | 11.4 | too high^*^ | too high^*^ | 40.6 | 4.94 | 1.41 | 2.3 | 7.23 | 13.3 | 3.77 | 0.064 | 12.001 | too low^*^ |

^*^ Outside the range of the measuring instrument.
